# Survival after bilateral risk-reducing mastectomy in healthy *BRCA1* and *BRCA2* mutation carriers

**DOI:** 10.1007/s10549-019-05345-2

**Published:** 2019-07-13

**Authors:** Bernadette A. M. Heemskerk-Gerritsen, Agnes Jager, Linetta B. Koppert, A. Inge-Marie Obdeijn, Margriet Collée, Hanne E. J. Meijers-Heijboer, Denise J. Jenner, Hester S. A. Oldenburg, Klaartje van Engelen, Jakob de Vries, Christi J. van Asperen, Peter Devilee, Marinus J. Blok, C. Marleen Kets, Margreet G. E. M. Ausems, Caroline Seynaeve, Matti A. Rookus, Maartje J. Hooning

**Affiliations:** 1 Department of Medical Oncology, Erasmus MC Cancer Institute, PO Box 5201, 3008 AE Rotterdam, The Netherlands; 2 Department of Surgery, Erasmus MC Cancer Institute, Rotterdam, The Netherlands; 3 Department of Radiology, Erasmus MC Cancer Institute, Rotterdam, The Netherlands; 4 Department of Clinical Genetics, Erasmus MC Cancer Institute, Rotterdam, The Netherlands; 5 Department of Clinical Genetics, Academic Medical Center, Amsterdam, The Netherlands; 6 Department of Epidemiology, Netherlands Cancer Institute-Antoni van Leeuwenhoek Hospital, Amsterdam, The Netherlands; 7 Department of Surgery, Netherlands Cancer Institute, Amsterdam, The Netherlands; 8 Department of Clinical Genetics, VU University Medical Center, Amsterdam, The Netherlands; 9 Department of Surgery, University Medical Center Groningen, Groningen, The Netherlands; 10 Department of Clinical Genetics, Leiden University Medical Center, Leiden, The Netherlands; 11 Department of Human Genetics, Leiden University Medical Center, Leiden, The Netherlands; 12 Department of Clinical Genetics, Maastricht University Medical Center, Maastricht, The Netherlands; 13 Department of Human Genetics, Radboud University Medical Center, Nijmegen, The Netherlands; 14 Department of Medical Genetics, University Medical Center Utrecht, Utrecht, The Netherlands

**Keywords:** *BRCA1/2*, Bilateral risk-reducing mastectomy, Prevention, Surveillance, Survival

## Abstract

**Background:**

In healthy *BRCA1/2* mutation carriers, bilateral risk-reducing mastectomy (BRRM) strongly reduces the risk of developing breast cancer (BC); however, no clear survival benefit of BRRM over BC surveillance has been reported yet.

**Methods:**

In this Dutch multicenter cohort study, we used multivariable Cox models with BRRM as a time-dependent covariable to estimate the associations between BRRM and the overall and BC-specific mortality rates, separately for *BRCA1* and *BRCA2* mutation carriers.

**Results:**

During a mean follow-up of 10.3 years, 722 out of 1712 *BRCA1* (42%) and 406 out of 1145 *BRCA2* (35%) mutation carriers underwent BRRM. For *BRCA1* mutation carriers, we observed 52 deaths (20 from BC) in the surveillance group, and 10 deaths (one from BC) after BRRM. The hazard ratios were 0.40 (95% CI 0.20–0.90) for overall mortality and 0.06 (95% CI 0.01–0.46) for BC-specific mortality. BC-specific survival at age 65 was 93% for surveillance and 99.7% for BRRM. For *BRCA2* mutation carriers, we observed 29 deaths (7 from BC) in the surveillance group, and 4 deaths (no BC) after BRRM. The hazard ratio for overall mortality was 0.45 (95% CI 0.15–1.36). BC-specific survival at age 65 was 98% for surveillance and 100% for BRRM.

**Conclusion:**

BRRM was associated with lower mortality than surveillance for *BRCA1* mutation carriers, but for *BRCA2* mutation carriers, BRRM may lead to similar BC-specific survival as surveillance. Our findings support a more individualized counseling based on BRCA mutation type.

**Electronic supplementary material:**

The online version of this article (10.1007/s10549-019-05345-2) contains supplementary material, which is available to authorized users.

## Introduction

Women with a germline *BRCA1/2* gene mutation have high risks of developing breast cancer (BC), estimated to range from 45 to 88% for a first BC up to the age of 70 years [1–4]. Moreover, BC is diagnosed at a younger age in *BRCA1/2* mutation carriers than in the general population [4–6], with an increased risk from the age of 25 years. For healthy *BRCA1/2* mutation carriers, the options are to follow a BC surveillance program aimed at early BC detection, or to opt for bilateral risk-reducing mastectomy (BRRM) to reduce BC risk. In healthy *BRCA1/2* mutation carriers, BRRM reduces the risk of BC with estimates even up to 100% [7–12], and this method may have beneficial effects on quality of life by diminishing the strong anxiety of getting BC. However, despite the strong BC risk-reduction, no clear survival benefit of BRRM over BC surveillance has been reported so far.

Mathematical models with simulated cohorts suggested that surveillance with both mammography and magnetic resonance imaging (MRI) in combination with risk-reducing salpingo-oophorectomy might offer an almost comparable survival as BRRM with risk-reducing salpingo-oophorectomy, due to improved imaging techniques and better systemic treatment options in recent years [13–15]. However, no convincing prospective data are available so far. Previously, we observed better 10-year overall survival in the BRRM group than in the surveillance group (99% vs. 96%) among 570 healthy *BRCA1/2* mutation carriers, but this difference was not significant [10].

To investigate whether BRRM leads to survival benefit, we determined the overall and breast cancer-specific mortality rates among 2857 healthy *BRCA1*/*2* mutation carriers opting for either BRRM or surveillance with follow-up until 2017. Since *BRCA2*-associated BCs have more favorable characteristics than *BRCA1*-associated BCs [10, 16, 17], and *BRCA2* mutation carriers have shown lower recurrence rates than *BRCA1* mutation carriers [10], we performed all analyses for *BRCA1* and *BRCA2* mutation carriers separately.

## Participants and methods

### Study population

In the context of the Hereditary Breast and Ovarian Cancer Netherlands (HEBON) study, members of breast and/or ovarian cancer families are being identified through the departments of Clinical Genetics/Family Cancer Clinics at eight Dutch academic centers and the Netherlands Cancer Institute [18]. Written informed consent was obtained from each participant, or from a close relative in case of already deceased individuals. As of January 1999, relevant data on participants, including data on preventive strategies, the occurrence of cancer and vital status, were retrieved and updated through medical files and questionnaires, and through linkages to the Netherlands Cancer Registry, the Dutch Pathology Database, and the municipal registry database. The latest follow-up date was December 31, 2016. The study was approved by the Medical Ethical Committees of all participating centers.

**Fig. 1:**
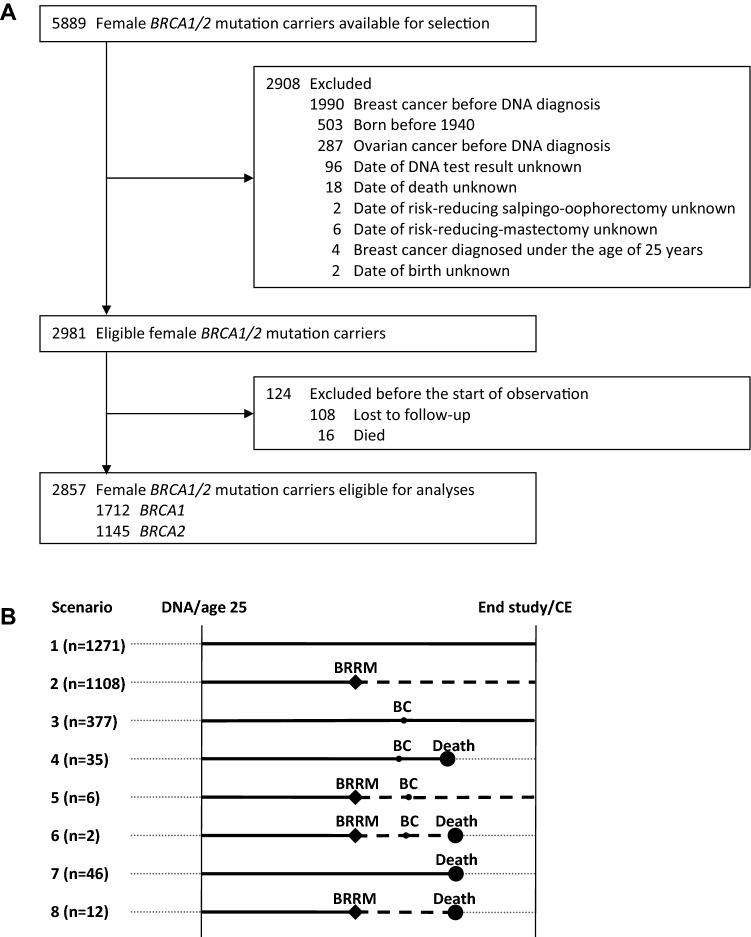
Flowchart of inclusion of participants (**a**) and Design of the analytic method and allocation of person-years of observation (**b**). *DNA* date of DNA test result, *CE* censoring event, *BRRM* bilateral risk-reducing mastectomy, *BC* first breast cancer. As visualized in **b**, observation started at the age at DNA test result, or age 25, whichever came last. For women not opting for BRRM, we allocated all person-years of observation (PYO) to the surveillance group (solid lines; scenarios 1, 3, 4, 7). For women opting for BRRM, we allocated PYO before surgery to the surveillance group, and PYO after surgery to the BRRM group (dashed lines; scenarios 2, 5, 6, and 8). The observation ended on the age of death (any cause), or age at study closing date (i.e., December 31, 2016), whichever came first

From this national cohort, we identified 5889 germline *BRCA1/2* mutation carriers. Women were eligible for the study if they had no history of cancer—to avoid cancer-induced testing bias [19, 20]—and had both breasts and both ovaries in situ at the date of DNA test result. As shown in Fig. 1a, we selected 1712 *BRCA1* and 1145 *BRCA2* mutation carriers.

### Data collection

We retrieved data on type of mutation (i.e., *BRCA1* or *BRCA2*) and date of DNA diagnosis; dates of birth, of diagnoses of first BC, ovarian cancer, and other cancers; dates of BRRM and risk-reducing salpingo-oophorectomy; and date and cause of death. We also collected data on BC characteristics (size, nodal status, behavior, differentiation grade, hormone receptor status, and HER2 status) and BC treatment details.

### Breast cancer surveillance for *BRCA1/2* mutation carriers in the Netherlands

BC surveillance for *BRCA1/2* mutation carriers consisted of annual imaging by MRI between 25 and 60 years (since 1998), next to annual imaging by mammography from 30 till 60 years of age, biennial (annual since 2012) mammography from age 60 till age 75, and annual clinical breast examination from the age of 25 years onward [21]. For the current cohort, the actual attendance to the surveillance program was derived from self-reported data.

### Statistical analyses

We evaluated person characteristics by comparing women who opted for BRRM (BRRM group) with women who did not until the end of follow-up (surveillance group). Differences between the BRRM and the surveillance group were tested by using *χ*^2^ for categorical variables, and the two-sample Wilcoxon rank-sum (Mann–Whitney) test for continuous variables.

The outcomes, overall mortality and breast cancer-specific mortality, were measured in person-years of observation. We started the observation period at the age at DNA test result or 25 years of age (since from this age regular BC surveillance is offered to Dutch *BRCA1/2* mutation carriers), whichever came last. Figure 1b depicts the allocation of person-years of observation to the BRRM and the surveillance group. For women who opted for BRRM and had unexpected malignant findings in the mastectomy specimens, we considered BC as being developed before BRRM, and therefore we allocated all person-years of observation to the surveillance group. The observation period ended at the age at last follow-up or death (due to all causes for the overall survival analyses and from BC for the breast cancer-specific analyses). The earliest date of DNA result was January 3, 1995.

To estimate the associations between BRRM and survival endpoints, we used extended Cox models with BRRM as a time-dependent variable to obtain hazard ratios (HRs) and accompanying 95% confidence intervals (CIs), using the surveillance group as the reference. To adjust for potential confounders, we generated a propensity score, based on year of birth, age at start of observation, age at DNA test result, year of DNA test result, and undergoing risk-reducing salpingo-oophorectomy (yes or no; time dependent) and performed multivariable analyses with the propensity score as covariable. For the mentioned variables all data were available for all participants. To graph the cumulative survival curves for the BRRM and the surveillance group, we used the Simon and Makuch method—which takes into account the change in an individual’s covariate status over time—with chronological age as the time variable [22, 23]. Using the log-rank test for equality of survivor functions, we tested whether the curves were significantly different from each other. We performed all analyses separately for *BRCA1* and *BRCA2* mutation carriers.

All *P* values were two-sided, and a significance level *α* = 0.05 was used. Analyses were performed using STATA (version 14.1, StataCorp, Collegestation, TX).

## Results

### Study population

Of the 1712 selected *BRCA1* mutation carriers, 722 opted for BRRM, and 406 of the 1145 *BRCA2* mutation carriers opted for BRRM (Table 1). Women opting for BRRM underwent DNA testing at a younger age than women who stayed under surveillance until end of follow-up (median age 34 vs. 38 for *BRCA1*, and 36 vs. 42 for *BRCA2* mutation carriers). Also, women in the BRRM group more often opted for risk-reducing salpingo-oophorectomy than women in the surveillance group [557 (77%) vs. 569 (57%) for *BRCA1*, and 293 (72%) vs. 441 (60%) for *BRCA2* mutation carriers] at a younger age (median age 40 vs. 44 for *BRCA1*, and 42 vs. 47 for *BRCA2* mutation carriers; Table 1).

**Table 1:** Characteristics of *BRCA1* and *BRCA2* mutation carriers at risk of breast cancer

	*BRCA1* mutation carriers			*BRCA2* mutation carriers		
	BRRM	Surveillance	*P* value^a^	BRRM	Surveillance	*P* value^a^
*N* (%)	722 (42%)	990 (58%)		406 (35%)	739 (65%)	
Observation period, median years (IQR)	10.6 (7.9–15.4)	9.3 (6.7–13.3)	< 0.001	9.9 (7.1–12.5)	8.6 (6.5–11.7)	< 0.001
Observation period after BRRM, median years (IQR)	8.5 (5.5–12.9)	–		7.2 (4.8–10.8)	–	
Age at start of observation, median years (IQR)	34 (29–41)	38 (30–47)	< 0.001	36 (29–43)	42 (33–51)	< 0.001
DNA test result						
Median age (IQR)	34 (29–41)	38 (30–47)	< 0.001	36 (29–43)	42 (33–51)	< 0.001
Median year (IQR)	2006 (2001–2008)	2007 (2003–2009)	< 0.001	2006 (2004–2009)	2008 (2004–2010)	< 0.001
Year of birth						
1940–1949	16 (2%)	87 (9%)	< 0.001	11 (3%)	92 (13%)	< 0.001
1950–1959	96 (13%)	199 (20%)		62 (15%)	170 (23%)	
1960–1969	236 (33%)	288 (29%)		128 (32%)	210 (28%)	
1970–1979	268 (37%)	242 (24%)		140 (34%)	177 (24%)	
> 1980	106 (15%)	174 (18%)		65 (16%)	90 (12%)	
Median (IQR)	1970 (1963–1976)	1967 (1958–1975)	0.002	1970 (1962–1977)	1966 (1955–1974)	< 0.001
BRRM	722 (100%)	–		406 (100%)	–	
Median age (IQR)	37 (32–43)	–		38 (33–45)	–	
Median year (IQR)	2008 (2003–2011)	–		2009 (2006–2012)	–	
RRSO, *n*/*N* (%)	557/722 (77%)	569/990 (57%)	< 0.001	293/406 (72%)	441/739 (60%)	< 0.001
Median age (IQR)	40 (37–44)	44 (40–51)	< 0.001	42 (39–48)	47 (42–55)	< 0.001
Median year (IQR)	2008 (2004–2011)	2008 (2005–2011)	0.849	2009 (2006–2011)	2009 (2006–2011)	0.535
Before/with/after BRRM	258/64/235	–		146/22/125	–	
Breast cancer, *n*/*N* (%)	8/722 (1%)	268/990 (27%)^b^	< 0.001	0/406 (0%)	144/739 (19%)^c^	< 0.001
Median age (IQR)	45 (34–48)	44 (35–50)	0.781	–	48 (39–55)	NA
Median year (IQR)	2010 (2006–2012)	2009 (2005–2011)	0.953	–	2010 (2007–2013)	NA
Median years after BRRM (IQR)	4.4 (1.0–6.6)	–		–	–	
CRRM after breast cancer	–	172 (17%)		–	97 (13%)	
Ovarian cancer, *n*/*N* (%)	16/722 (2%)	34/990 (3%)	0.139	4/406 (1%)	15/739 (2%)	0.186
Median age (IQR)	45 (38–53)	50 (43–56)	0.169	45 (39–52)	54 (51–63)	0.072
Median year (IQR)	2006 (2002–2011)	2008 (2003–2011)	0.392	2013 (2011–2015)	2009 (2007–2011)	0.063
Before/after BRRM	8/8	–		1/3	–	
Other tumor (no OC or BC), *n*/*N* (%)	47/722 (7%)	67/990 (7%)	0.833	23/406 (6%)	66/739 (9%)	0.048
Median age (IQR)	47 (39–53)	54 (42–62)	0.005	47 (36–50)	52 (41–62)	0.009
Before/after BRRM	16/31	–		7/16	–	
Death (all causes), *n*/*N* (%)	10/722 (1%)	52/990 (5%)	< 0.001	4/406 (1%)	29/739 (4%)	0.004
Median age (IQR)	53 (47–63)	53 (44–58)	0.293	55 (52–58)	61 (52–67)	0.205
Median year (IQR)	2014 (2011–2015)	2009 (2006–2013)	0.011	2011 (2009–2014)	2011 (2009–2014)	0.846
Cause of death^d^, *n*/*N* (%)						
Breast cancer	1/10 (10%)	20/52 (38%)	0.241	0/4 (0%)	7/29 (24%)	0.635
Ovarian cancer	5/10 (50%)	19/52 (37%)		0/4 (0%)	2/29 (7%)	
Other malignancy	4/10 (40%)^e^	13/52 (25%)^f^		3/4 (75%)^g^	15/29 (52%)^h^	
Not due to malignancy	0/10 (0%)	0/52 (0%)		1/4 (25%)	5/29 (17%)	

*BRRM* bilateral risk-reducing mastectomy, *IQR* interquartile range, *RRSO* risk-reducing salpingo-oophorectomy, *n*/*N* number out of total number of women with nonmissing data on the variable of interest, *OC* ovarian cancer, *BC* breast cancer, *CRRM* contralateral risk-reducing mastectomy

^a^Differences between the BRRM and the surveillance groups were tested by using *χ*^2^ for categorical variables, and the two-sample Wilcoxon rank-sum (Mann–Whitney) test for continuous variables

^b^*N *= 27 found as unexpected malignant finding in the mastectomy specimens of women initially opting for BRRM. Three of these patients died during the observation period; two of BC, one of another malignancy

^c^*N *= 13 found as unexpected malignant finding in the mastectomy specimens of women initially opting for BRRM. None of these patients died during the observation period

^d^Retrieved from the Netherlands Cancer Registry (44%) or derived from the available data on recurrent disease and ages at cancer diagnoses and death (56%)

^e^Stomach (*N *= 1), pancreas (*N *= *2*), lymph nodes (*N *= 1)

^f^Esophagus (*N *= 1), rectum/rectosigmoid (*N *= 2), bialiary tract (*N *= 1), pancreas (*N *= 2), lung (*N *= 3), bone marrow (*N *= 1), skin (*N *= 1), brain (*N *= 1), unknown primary site (*N *= 1)

^g^Pancreas (*N *= 2), lung (*N *= 1)

^h^Colon (*N *= 2), bialiary tract (*N *= 1), pancreas (*N *= 7), lung (*N *= 3), skin (*N *= 1), bladder (*N *= 1)

### Breast cancer

BC occurrence (including both invasive and ductal carcinoma in situ) was lower in the BRRM than in the surveillance group [8 (1%) vs. 268 (27%) for *BRCA1*, and 0 (0%) vs. 144 (19%) for *BRCA2* mutation carriers; Table 1]. Among *BRCA1* mutation carriers, we observed no differences in tumor characteristics of BCs occurring after BRRM and during surveillance (see Supplementary Table S1).

As shown in Table 2, *BRCA2*-associated BCs were diagnosed with more favorable characteristics than *BRCA1*-associated BCs, i.e., diagnosed at older age, more often in situ, better differentiated, and less often showing a triple-negative phenotype. Consequently, *BRCA2* mutation carriers were less often treated with chemotherapy, and more often treated with endocrine therapy.

**Table 2:** *BRCA1*- and *BRCA2*-associated breast cancer characteristics and therapy

	Germline gene mutation		
	*BRCA1*	*BRCA2*	*P* value^a^
*N*	276 (16%)	144 (13%)	0.009
Age at diagnosis, median (IQR)	44 (35–50)	48 (39–55)	< 0.001
Detection, *n*/*N* (%)			
Symptoms	30/121 (25%)	11/46 (24%)	0.607
Screen-detected	77/121 (63%)	27/46 (59%)	
Detected between 2 screening rounds	14/121 (12%)	8/46 (17%)	
Behavior, *n*/*N* (%)			
In situ^b^	32/270 (12%)	36/143 (25%)	0.001
Invasive	238/270 (88%)	107/143 (75%)	
Bloom and Richardson differentiation grade, *n/N* (%)			
I	9/251 (3%)	11/126 (9%)	< 0.001
II	57/251 (23%)	68/126 (54%)	
III	185/251 (74%)	47/126 (37%)	
pT-status, *n*/*N* (%)			
0 (in situ)	32/253 (13%)	36/136 (26%)	0.001
1	163/253 (64%)	79/136 (58%)	
2	58/253 (23%)	19/136 (14%)	
3	0/253 (0%)	2/136 (2%)	
pN-status, *n*/*N* (%)			
0	207/248 (84%)	100/129 (78%)	0.164
1	33/248 (13%)	25/129 (19%)	
2	6/248 (2%)	1/129 (1%)	
3	2/248 (1%)	3/129 (2%)	
Positive ER-status^c^, *n*/*N* (%)	49/224 (22%)	79/101 (78%)	< 0.001
Positive PR-status^c^, *n*/*N* (%)	36/219 (16%)	55/100 (55%)	< 0.001
Positive Her2-status^d^, *n*/*N* (%)	10/170 (6%)	8/75 (11%)	0.186
Triple-negative^e^, *n*/*N* (%)	128/166 (77%)	17/75 (23%)	< 0.001
Treatment primary breast cancer			
Chemotherapy	170/273 (62%)	56/142 (39%)	< 0.001
Endocrine therapy	42/262 (16%)	51/134 (38%)	< 0.001
Targeted therapy	14/263 (5%)	4/134 (3%)	0.290
Mastectomy	204/261 (78%)	110/134 (82%)	0.360
Radiotherapy	64/263 (24%)	38/134 (28%)	0.386

*IQR* interquartile range; *n*/*N* number out of total number of women with nonmissing data on the variable of interest, *ER* estrogen receptor, *PR* progesterone receptor

^a^Differences between *BRCA1*- and *BRCA2*-associated breast cancers were tested by using *χ*^2^ for categorical variables, and the two-sample Wilcoxon rank-sum (Mann–Whitney) test for continuous variables

^b^All diagnosed in the surveillance groups

^c^Hormone receptors were considered positive if staining was seen in ≥ 10% of the nuclei, according to the Dutch national guidelines for breast cancer treatment

^d^Her2 receptor status was scored according to international guidelines. An equivocal immunohistochemical result (2+) was followed by fluorescence in situ hybridization

^e^ER-negative, PR-negative, and Her2-negative

### Overall mortality

**Fig. 2:**
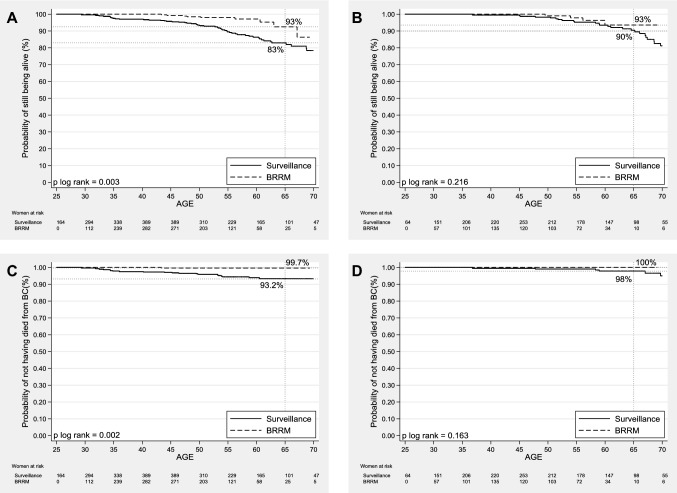
Overall survival curves for *BRCA1* (**a**) and *BRCA2* (**b**) mutation carriers and breast cancer-specific survival curves for *BRCA1* (**c**) and *BRCA2* (**d**) mutation carriers opting for bilateral risk-reducing mastectomy (BRRM) versus staying under surveillance, using the Simon and Makuch method—which takes into account the change in an individual’s variable status over time—with chronological age as the time variable

All-cause mortality rates were lower for women opting for BRRM than for women under surveillance (Table 3). For *BRCA1* mutation carriers, the multivariable Cox model yielded an HR of 0.40 (95% CI 0.20–0.80) in favor of the BRRM group. The unadjusted survival curves showed a probability of being alive at 65 years of 93% for the BRRM group and 83% for the surveillance group (Fig. 2a). For *BRCA2* mutation carriers, the multivariable HR was 0.45 (95% CI 0.15–1.36) (Table 3), and the probability of being alive at the age of 65 was 93% the BRRM group, and 90% for the surveillance group (Fig. 2b).

**Table 3:** Associations of bilateral risk-reducing mastectomy with all-cause mortality and breast cancer-specific mortality

	*BRCA1* mutation carriers		*BRCA2* mutation carriers	
	BRRM	Surveillance	BRRM	Surveillance
PYO	6647	11,782	3225	7808
All-cause mortality				
Deaths	10	52	4	29
All-cause mortality rate (95% CI)^a^	1.5 (0.8–2.8)	4.4 (3.4–5.8)	1.2 (0.4–3.3)	3.7 (2.6–5.3)
HR (95% CI)^b^	0.37 (0.19–0.73)		0.52 (0.18–1.50)	
HR (95% CI)^c^	0.40 (0.20–0.80)		0.45 (0.15–1.36)	
Breast cancer-specific mortality				
Deaths due to BC	1	20	0	7
BC-specific mortality rate (95% CI)^a^	0.2 (0.02–1.1)	1.7 (1.1–2.6)	0	0.9 (0.4–1.9)
HR (95% CI)^b^	0.08 (0.01–0.62)		NA	
HR (95% CI)^c^	0.06 (0.01–0.46)		NA	

*BRRM* bilateral risk-reducing mastectomy, *PYO* person-years of observation, *HR* (95% CI), hazard ratio (95% confidence interval), *BC* breast cancer, *NA* not applicable

^a^Per 1000 PYO

^b^Univariable

^c^Multivariable, adjusted for the estimated propensity score, which was based on year of birth, age at start of observation, and undergoing RRSO (yes or no; time dependent)

### Breast cancer-specific mortality

Breast cancer-specific mortality rates were lower for women opting for BRRM than for women under surveillance (Table 3). Eventually, one *BRCA1* (0.1%) and no *BRCA2* mutation carriers died due to BC after BRRM, while from the surveillance group 20 *BRCA1* (2.0%) and 7 *BRCA2* (0.9%) mutation carriers died due to BC. For *BRCA1* mutation carriers, the multivariable HR was 0.06 (95% CI 0.01–0.46) in favor of the BRRM group. At the age of 65, the probability of not having died due to BC was 99.7% for the BRRM group and 93% for the surveillance group (Fig. 2c). For *BRCA2* mutation carriers, no HR could be estimated, as not one woman opting for BRRM died due to BC (Table 3). The probability of not having died due to BC at the age of 65 was 100% in the BRRM and 98% in the surveillance group (Fig. 2d).

## Discussion

In this nationwide cohort study, we observed lower overall and breast cancer-specific mortality rates among *BRCA1* mutation carriers opting for BRRM than among those under surveillance. For *BRCA2* mutation carriers, BRRM was nonsignificantly associated with lower overall mortality when compared with surveillance. Not one *BRCA2* mutation carrier died of BC after BRRM, while the surveillance group performed almost as good. In addition, *BRCA2*-associated BCs were diagnosed less frequently, and had more favorable characteristics than *BRCA1*-associated BCs.

All analyses were performed separately for *BRCA1* and *BRCA2* mutation carriers, which is more accurate because *BRCA1*-associated BCs and *BRCA2*-associated BCs represent different entities. The current results are in line with our previous observation of a small but nonsignificant better 10-year overall survival after BRRM than under surveillance (99% vs. 96%) for a smaller combined cohort of *BRCA1/2* mutation carriers [10]. The observation that BRRM was associated with lower breast cancer-specific mortality for *BRCA1* mutation carriers, and not for *BRCA2* mutation carriers underscores that counseling for *BRCA1* and *BRCA2* mutation carriers regarding the choice between risk-reducing mastectomy and surveillance might be tailored, although confirmation in a larger cohort of especially *BRCA2* mutation carriers is warranted.

To the best of our knowledge, this is the first cohort study comparing BRRM with surveillance with respect to survival in healthy *BRCA1* and *BRCA2* mutation carriers separately. Previous investigations have shown that BRRM effectively reduces BC risk [7–12, 24, 25], but convincing data regarding survival after BRRM in *BRCA1/2* mutation carriers are scarce and mainly derived from modeling studies. Using a simulated cohort and Markov modeling of outcomes, Grann et al. estimated that BRRM plus risk-reducing salpingo-oophorectomy at the age of 30 may extend survival by 4.9 years over surveillance alone [26]. Further, Sigal et al. yielded from their Monte Carlo simulation model gains in life expectancy after BRRM plus risk-reducing salpingo-oophorectomy varying from 6.8 to 10.3 for *BRCA1* and 3.4 to 4.4 years for *BRCA2* mutation carriers [15]. Recently, Giannakeas and Narod showed in a simulated cohort that for *BRCA* mutation carriers who underwent bilateral mastectomy at the age of 25, the probability of being alive at age 80 increased by 8.7% [27]. In addition, in an exploratory study in unaffected *BRCA1/2* mutation carriers and untested female first-degree relatives, Ingham et al. showed overall survival benefit of ~ 10% after risk-reducing surgery [28]. However, this study is not directly comparable to the current study since the authors compared three groups of women undergoing risk-reducing surgery (i.e., BRRM only, risk-reducing salpingo-oophorectomy only, or both) with women without any surgery, while we currently incorporated undergoing risk-reducing salpingo-oophorectomy (yes/no) in the model. In our opinion, this better reflects daily practice: as a result of directive counseling due to ineffective screening protocols for early ovarian cancer detection, the uptake of risk-reducing salpingo-oophorectomy is high for both women undergoing BRRM (~ 75%) and women not (yet) opting for BRRM (~ 60%).

For *BRCA1* mutation carriers under surveillance, BC and ovarian cancer were the main causes of death. The high percentage of ovarian cancer deaths in this group—which was similar to that of BC deaths—emphasizes the need for RRSO for BRCA mutation carriers. While in the surveillance group 20 out of 990 women (2.0%) died due to BC, only one out of 722 women (0.1%) died from BC after BRRM. The latter patient was identified with a *BRCA1* mutation at the age of 38, and underwent BRRM 1 year later (in 2007). At the age of 42, she was diagnosed with a triple-negative BC with lung metastases, and died 1 year later. This emphasizes—in addition to the fact that eight BCs occurred 4.4 median years after BRRM in the current cohort—that BRRM does not fully protect against the occurrence of BC and BC-related death.

Of the 29 deceased *BRCA2* mutation carriers in the surveillance group, 24% died of BC, 59% of another malignancy—including two deaths due to ovarian cancer and seven due to pancreatic cancer—and 17% died of nonmalignancy-related causes. The higher numbers of non BC-related deaths in the surveillance group seem to be coincidental, but may explain the higher overall mortality rate though comparable breast cancer-specific mortality rate among *BRCA2* mutation carriers under surveillance.

In *BRCA2* mutation carriers, we observed no BCs and no BC-related deaths after BRRM versus 144 BC cases and seven BC-related deaths in the surveillance group, suggesting a maximal risk-reduction of developing BC and dying due to BC after BRRM. However, the absolute breast cancer-specific survival benefit at the age of 65 was minimal (2%), partly due to the low BC-specific mortality in the surveillance group (i.e., 0.9 per 1000 person-years of observation). The latter can be explained by the observation that *BRCA2*-associated BCs were diagnosed with more favorable characteristics, i.e., diagnosed at older age, more often in situ, better differentiated, and less often showing a triple-negative phenotype—than *BRCA1*-associated BCs. This supports previous suggestions that *BRCA2*-associated BC patients face a better prognosis than *BRCA1*-associated BC patients [10, 16]. The current results suggest that regarding breast cancer-specific mortality, BC surveillance may be a reasonable and balanced alternative to BRRM for *BRCA2* mutation carriers.

The main strengths of the current study are (1) the sufficient numbers of *BRCA1* and *BRCA2* mutation carriers allowing analyses for both groups separately, (2) with long enough follow-up, and (3) the availability of data on cause of death, enabling to specifically address the ultimate goal for BRRM, i.e., breast cancer-specific survival.

This study also has limitations. First, information regarding BC screening modality and frequency was derived from self-reported data, and unknown for ~ 50% of the women in the surveillance groups. However, we do know that all women had been counseled by clinical geneticists and were aware of an identified *BRCA* mutation at the start of the observation period. Therefore, we assume that the vast majority of the women did participate in a BC surveillance program for high-risk women according to Dutch guidelines. This assumption is supported by the experience from the Rotterdam Family Cancer Clinic that after being positively tested for a pathologic mutation in one of the BRCA genes, 97% of the mutation carriers is yearly screened; 79% of the mutation carriers are yearly screened by both MRI and mammography, 11% by MRI only (aged < 30 years), and 7% by mammography only (aged > 60 years). Only three percent of the proven mutation carriers seem not to attend the national screening program for *BRCA1/2* mutation carriers, or are screened in another hospital (unpublished data). These numbers are in line with recently reported international trends in the uptake of cancer screening among *BRCA1/2* mutation carriers [29].

Still, if the BC patients with unknown screening status were not under BC surveillance, BCs consequently would be diagnosed at a more advanced stage with worse prognosis. As a result, the observed number of BC-related deaths in the surveillance group could be an overestimation of the actual number of BC-related deaths under surveillance, and a potential breast cancer-specific survival benefit may be overestimated. However, BCs occurring among *BRCA1/2* mutation carriers in the surveillance group with unknown screening status showed in fact slightly more favorable characteristics (i.e., more often in situ and smaller than two centimeters; see Supplementary Table S2) than the patients with known screening status. In addition, the absolute number of women dying from BC was lower among the women with unknown screening status: 8 out 864 (0.9%) versus 19 out of 865 (2.2%) among the women with known screening status (*P* value 0.033; Supplementary Table S2). Thus, it seems plausible that the majority of the women with unknown screening status were actually under BC surveillance, and an overestimation of the observed breast cancer-specific survival is unlikely.

A second limitation may be that family history is not available for all participants. If all women from families with high risks of developing BC—usually at young age—opt for BRRM, this may lead to an overrepresentation of women with lower family-based BC risks in the surveillance groups. Subsequently, the baseline BC risk and following BC-specific mortality may be underestimated in the surveillance groups, leading to an underestimation of potential survival benefit after BRRM. However, despite this potential underestimation, the study found an association with better breast cancer-specific survival for *BRCA1* mutation carriers after BRRM. Still, as the influence of family history cannot be ruled out, it will be interesting to take family history into account in future studies.

Thirdly, there might be some bias toward BRRM being offered more often to healthier women. This could be supported by the fact that *BRCA2* mutation carriers in the surveillance group show more other cancers (i.e., no BC or ovarian cancer) than those in the BRRM group (9% vs. 6%, *P* = 0.048; Table 1). However, we did not observe this difference for *BRCA1* mutation carriers, where the incidence of other tumors was 7% for both groups. In addition, the median age at diagnosis of cancer other than BC or ovarian cancer is higher in the surveillance group than in the BRRM group (both for *BRCA1* and *BRCA2* mutation carriers), suggesting that with longer follow-up—and thus growing age—the numbers of patients with other tumors could increase. Unfortunately, data about health-related issues such as weight and past and current smoking habits are not available for the current cohort.

In conclusion, BRRM was associated with lower overall and breast cancer-specific mortality rates than surveillance for *BRCA1* mutation carriers. For *BRCA2* mutation carriers, BRRM may lead to similar breast cancer-specific survival as surveillance. The latter is most probably due to the more favorable characteristics of *BRCA2*-associated BCs. Therefore, for *BRCA2* mutation carriers BC surveillance may be as effective as BRRM regarding breast cancer-specific survival. Although the number of events are small—especially for the analyses on breast cancer-specific mortality—our findings may support a more individualized counseling based on BRCA mutation type regarding the difficult choice between BRRM and BC surveillance.

## Electronic supplementary material

Below is the link to the electronic supplementary material.

Supplementary material 1 (PDF 70 kb)
