# Supplementary material for: Survival after bilateral risk-reducing mastectomy in healthy BRCA1 and BRCA2 mutation carriers
Source: Breast Cancer Res Treat. 2019 Jul 13;177(3):723–33. doi: 10.1007/s10549-019-05345-2 (PMC6745043; doi:10.1007/s10549-019-05345-2)
Supplement: Supplementary file 1 — Supplementary material 1 (PDF 70 kb) [file 10549_2019_5345_MOESM1_ESM.pdf]

Online-only material accompanying manuscript entitled “Survival after bilateral risk-reducing mastectomy in healthy *BRCA1* and *BRCA2* mutation carriers”

Breast Cancer Research and Treatment

Corresponding author

Dr. Bernadette A.M. Heemskerk-Gerritsen

Erasmus MC Cancer Institute

Department of Medical Oncology

PO Box 5201

3008 AE Rotterdam

The Netherlands

Phone +31 10 7035484

b.heemskerk-gerritsen@erasmusmc.nl

Including:

- Table S1. *BRCA1*-associated breast cancer characteristics
- Table S2. Differences between *BRCA1/2* mutation carriers (combined) with known and unknown screening status in the surveillance group

Table S1. *BRCA1*-associated breast cancer characteristics

	BRRM	Surveillance	*P*-value[a]
**N**	8 (3%)	268 (97%)	
**Age at diagnosis, median (IQR)**	45 (34 - 48)	44 (35 - 50)	0.781
**Behavior, *n/N* (%)**			
**In situ**	0/7 (0%)	32/263 (12%)	0.326
**Invasive**	7/7 (100%)	231/263 (88%)	
**Bloom & Richardson differentiation grade, *n/N* (%)**			
**I**	0/6 (0%)	9/245 (4%)	0.753
**II**	2/6 (33%)	55/245 (22%)	
**III**	4/6 (67%)	181/245 (74%)	
**pT-status, *n/N* (%)**			
**0 (*in situ*)**	0/5 (0%)	32/248 (13%)	0.652
**1**	4/5 (80%)	159/248 (64%)	
**2**	1/5 (20%)	57/248 (23%)	
**pN-status, *n/N* (%)**			
**0**	5/6 (83%)	202/242 (83%)	0.969
**1**	1/6 (17%)	32/242 (13%)	
**2**	0/6 (0%)	6/242 (3%)	
**3**	0/6 (0%)	2/242 (1%)	
**Positive ER-status[b], *n/N* (%)**	2/6 (33%)	47/218 (22%)	0.491
**Positive PR-status[b], *n/N* (%)**	1/6 (17%)	35/213 (16%)	0.988
**Positive Her2-status[c], *n/N* (%)**	0/4 (0%)	10/166 (6%)	0.613
**Triple-negative[d], *n/N* (%)**	2/4 (50%)	126/160 (78%)	0.191

Abbreviations: BRRM, bilateral risk-reducing mastectomy; IQR, interquartile range; *n/N*, number out of total number of women with non-missing data on the variable of interest; ER, estrogen receptor; PR, progesterone receptor.

[a] Differences between the BRRM and the surveillance group were tested by using Chi-square for categorical variables, and the two-sample Wilcoxon rank-sum (Mann-Whitney) test for continuous variables

[b] Hormone receptors were considered positive if staining was seen in ≥10% of the nuclei, according to the Dutch national guidelines for breast cancer treatment.

[c] Her2 receptor status was scored according to international guidelines. An equivocal immunohistochemical result (2+) was followed by fluorescence in situ hybridization.

[d] ER-negative, PR-negative and Her2-negative.

Table S2. Differences between *BRCA1/2* mutation carriers (combined) with known and unknown screening status in the surveillance group

	Screening status		
	Known	Unknown	*P*-value[a]
N	**865 (50%)**	**864 (50%)**	
**Had screening[b], *n*/N (%)**	761/865 (88%)	-	
**Screening modality[b]**			
**Mx and MRI, *n*/N (%)**	537/761 (71%)	-	
**MRI only, *n*/N (%)**	102/761 (13%)	-	
**Mx only, *n*/N (%)**	74/761 (10%)	-	
**Unknown, *n*/N (%)**	48/761 (6%)	-	
**<u>Breast cancer</u>, *n/N* (%)**	249/865 (29%)	163/864 (19%)	<0.001
**Age at diagnosis, median years (IQR)**	43 (35 - 50)	46 (38 - 53)	0.035
**Behavior, *n/N* (%)**			
**In situ**	31/247 (13%)	37/159 (23%)	0.005
**Invasive**	216/247 (87%)	122/159 (77%)	
**Bloom & Richardson differentiation grade, *n/N* (%)**			
**I**	14/230 (6%)	6/141 (4%)	0.875
**II**	76/230 (33%)	47/141 (33%)	
**III**	140/230 (61%)	88/141 (63%)	
**pT-status, *n/N* (%)**			
**0**	31/239 (13%)	37/145 (25%)	0.013
**1**	157/239 (66%)	81/145 (56%)	
**2**	49/239 (20%)	27/145 (19%)	
**3**	2/239 (1%)	0/145 (0%)	
**pN-status, *n/N* (%)**			
**0**	189/231 (82%)	113/140 (81%)	0.412
**1**	34/231 (15%)	23/140 (16%)	
**2**	6/231 (2%)	1/140 (1%)	
**3**	2/231 (1%)	3/140 (2%)	
**Positive ER-status[c], *n/N* (%)**	80/210 (38%)	46/109 (42%)	0.477
**Positive PR-status[c], *n/N* (%)**	54/208 (26%)	36/105 (34%)	0.124
**Positive Her2-status[d], *n/N* (%)**	10/158 (6%)	8/84 (10%)	0.516
**Triple-negative[e], *n/N* (%)**	97/156 (62%)	46/81 (57%)	0.421
**<u>Treatment primary breast cancer</u>, *n/N* (%)**			
**Chemotherapy**	140/245 (57%)	80/162 (49%)	0.124
**Endocrine therapy**	55/241 (23%)	36/149 (24%)	0.761
**Targeted therapy**	8/241 (3%)	8/149 (5%)	0.321
**Radiotherapy**	64/241 (27%)	35/149 (23%)	0.499
**<u>Ovarian cancer</u>, *n/N* (%)**	21/865 (2%)	28/864 (3%)	0.308
**<u>Death</u>, *n/N* (%)**	34/865 (4%)	47/864 (5%)	0.138
**Cause of death, *n/N* (%)**			
**Breast cancer**	19/865 (2.2%)	8/864 (0.9%)	0.033
**Ovarian cancer**	7/865 (0.8%)	14/864 (1.6%)	0.124
**Other malignancy**	8/865 (0.9%)	20/864 (2.3%)	0.022
**Not due to malignancy**	0/865 (0%)	5/864 (0.8%)	0.008

Abbreviations: *n/N*, number out of total number of women with non-missing data on the variable of interest; Mx, mammography; MRI, magnetic resonance imaging; IQR, interquartile range; ER, estrogen receptor; PR, progesterone receptor.

[a] Differences between the groups were tested by using Chi-square for categorical variables, and the two-sample Wilcoxon rank-sum (Mann-Whitney) test for continuous variables

[b] Derived from self-reported data

[c] Hormone receptors were considered positive if staining was seen in ≥10% of the nuclei, according to the Dutch national guidelines for breast cancer treatment.

[d] Her2 receptor status was scored according to international guidelines. An equivocal immunohistochemical result (2+) was followed by fluorescence in situ hybridization.

[e] ER-negative, PR-negative and Her2-negative.
